# Arbeitsbedingte interstitielle Lungenerkrankungen

**DOI:** 10.1007/s00117-024-01342-9

**Published:** 2024-07-16

**Authors:** K. Hofmann-Preiß

**Affiliations:** Frankenstraße 24, 91096 Möhrendorf, Deutschland

**Keywords:** Silikose, Asbestose, Aluminose, Hartmetallfibrose, Low Dose HRCT, Silicosis, Asbestosis, Aluminosis, Hard metal lung disease, Low Dose HRCT

## Abstract

Eine Vielzahl von Expositionen am Arbeitsplatz (organische bzw. anorganischen Stäube, Gase, Rauche oder Dämpfe) kann eine diffuse interstitielle Lungenerkrankung (ILD) verursachen. Die Latenzzeit bis zum Auftreten der Erkrankung kann mehr als 30 Jahre betragen. Der Verlauf ist sehr unterschiedlich und hängt von der Menge der eingeatmeten Substanz und deren fibrogener Wirkung ab. Die pulmonalen Muster in der hochauflösenden Computertomographie (HRCT) unterscheiden sich nicht wesentlich von ILD anderer Ursachen. Ohne Kenntnis der beruflichen Vorgeschichte werden arbeitsbedingte ILD daher oft als idiopathisch eingestuft. Aus diesem Grund ist eine qualifizierte Berufsanamnese heute ein unverzichtbarer Bestandteil der interdisziplinären Diagnose von ILD.

## Abstract (en)

A variety of workplace exposures (organic or inorganic dusts as well as gases, fumes, or vapors) can cause diffuse interstitial lung disease. The latency period until onset of the disease can exceed 30 years. The disease course varies greatly and depends on the quantity of the inhaled substance and its fibrogenic effect. Pulmonary high-resolution computed tomography (HRCT) patterns do not differ significantly from those of interstitial lung diseases (ILD) of other etiologies. Therefore, without knowledge of the occupational history, work-related ILDs are often classified as idiopathic. In addition, there is increasing evidence in the recent literature that high exposure to silica dust can trigger autoimmune diseases (also involving the lungs). For this reason, a qualified occupational history is now an indispensable part of the interdisciplinary diagnosis of ILDs.

Arbeitsbedingte interstitielle Lungenerkrankungen (ILD) verursachen auch in modernen Industriegesellschaften noch immer eine erhebliche Krankheitslast. Sie werden durch die Inhalation von organischen/anorganischen Stäuben, Gasen, Rauchen oder Dämpfen verursacht. Bildmorphologisch können bei diesen Erkrankungen Muster aller ILD auftreten. Im Gegensatz zu den akuten/subakuten allergischen Reaktionen der Lunge werden die Staubspeichererkrankungen (klassische Pneumokoniosen) oft erst nach Latenzzeiten von mehr als 30 Jahren manifest. In Unkenntnis der Arbeitsanamnese werden sie dann nicht selten als idiopathisch eingestuft.

Arbeitsbedingte interstitielle Lungenerkrankungen verursachen auch in modernen Industriegesellschaften noch immer eine erhebliche Krankheitslast. Während der COVID-19-Pandemie standen arbeitsbedingte Atemwegsinfektionen im Vordergrund. Alleine in Deutschland wurden vom Pandemiebeginn bis Ende 2022 über 300.000 COVID-19-Erkrankungen als Berufskrankheit anerkannt. Nimmt man die Pandemie aus, bestehen die größten Risiken für die Entwicklung einer arbeitsbedingten interstitiellen Lungenerkrankung bei der Inhalation von Stäuben, Gasen und Dämpfen sowie Karzinogenen und Allergenen am Arbeitsplatz. Nach Schätzungen werden mehr als 10 % aller Lungenerkrankungen durch Schadstoffexpositionen am Arbeitsplatz verursacht [11].

Bei Arbeitsunfällen wird der Zusammenhang zwischen einer akut auftretenden ILD und einer arbeitsbedingten hohen Schadstoffexposition meist hergestellt.

Chronisch fibrosierende ILD, die durch eine langanhaltende niederschwellige Exposition entstehen und eine lange Latenzzeit zwischen Expositions- und Erkrankungsbeginn aufweisen, werden nicht selten als idiopathisch eingestuft, da sich Symptome und auch HRCT-Morphologie meist nicht von gleichartigen Erkrankungen andere Ursache unterscheiden und in der Routinediagnostik oft keine Arbeitsanamnese erhoben wird.

Blanc et al. [7] gehen davon aus, dass ca. 30 % der granulomatösen Lungenerkrankungen einschließlich der Sarkoidose, 29 % der Fälle mit Alveolarproteinose, 19 % der Fälle mit Hypersensitivitätspneumonitis und 26 % der als idiopathische pulmonale Fibrose (IPF) diagnostizierten pulmonalen Fibrosen eine arbeitsbedingte Ursache haben.

## Differenzierung arbeitsbedingter Erkrankungen – Berufskrankheiten

In Deutschland hat der Gesetzgeber bestimmte Erkrankungen, die durch arbeitsbedingte Expositionen entstehen unter Versicherungsschutz gestellt. Sie sind sie in der Anlage 1, sog. „Berufskrankheiten-Liste“, der Berufskrankheitenverordnung (BKV) [6] erfasst. Nur diese Erkrankungen werden in Deutschland als Berufskrankheiten (BK) bezeichnet. Nach §202 SGB-VII ist jeder Arzt, der den begründeten Verdacht auf eine solche Erkrankung äußert, verpflichtet diese unverzüglich anzuzeigen. (Die in der Berufskrankheiten-Liste aufgeführten Erkrankungen werden im Artikel deshalb auch mit BK-Nummer benannt.) Im Rahmen der Feststellung einer Berufskrankheit wird in Deutschland eine BMI-adaptierte Niedrigdosis-Computertomographie („low dose CT“, LDCT) gefordert [22]. Die Befundung erfolgt standardisiert nach ICOERD („International Classification of Occupational and Environmental Respiratory Diseases“; [14]). Die unfallversicherungsrechtliche Entscheidung beruht damit auf vergleichbaren und reproduzierbaren radiologischen Befunden.

## Arbeitsbedingte ILD mit akutem/subakutem Verlauf

*Organischer Staub, Gase, Chemikalien- oder Metalldämpfe bzw. -rauche* können bei erstmaliger Inhalation hoher Konzentrationen oder nach vorausgegangener Sensibilisierung eine akute ILD verursachen. Die schädigende Wirkung ist abhängig von der Löslichkeit und physikalisch-chemischen Eigenschaften des Stoffes. Eine Bronchiolitis, ein Lungenödem oder eine akuten ILD können die Folge sein (Tab. 1). Typischerweise treten Symptome akut bzw. mit einer Latenzzeit von einigen Stunden auf. Nach Expositionsende und frühzeitiger spezifischer Therapie sind die Erkrankungen meist reversibel.

**Tab. 1:** Radiologische Muster akut auftretender arbeitsbedingter interstitieller Lungenerkrankungen (ILD) und Beispiele für verursachende Schadstoffe. (Mod. nach [2])

Interstitielle Lungenerkrankung^a^	Verursachende Schadstoffe
Diffuser Alveolarschaden	Irritativ wirkende Gase, erhitzte Polymere, Paraquat, Zinkchlorid
Nichtkardiales Lungenödem	Irritativ wirkende Gase, erhitzte Polymere, Metalldämpfe
Nekrotisierende Bronchitis/Bronchiolitis	Irritativ wirkende Gase, Zinkchlorid, Petroleum
Organisierende Pneumonie	NO_2,_ Cadmium
Eosinophile Pneumonie	Nickel, Acetylen
Hypersensitivitätspneumonitis	Isocyanate, Beryllium, Antigene z. B. Pilzsporen, Proteine
Diffuse alveoläre Hämorrhagie (DAH)	Isocyanate, Anhydride

^a^Bildmorphologie s. bei jeweiliger ILD

Bildmorphologisch ist kein Rückschluss auf eine spezifische arbeitsbedingte Exposition möglich. Die qualifizierte Arbeitsanamnese ist entscheidend für die Zuordnung.

## Arbeitsbedingte chronisch verlaufende ILD

Mit Ausnahme des fibrosierenden Typs der Hypersensitivitätspneumonitis handelt es sich um Staubspeicherkrankheiten (Pneumokoniosen), die durch fibrogene anorganische Stäube (z. B. Quarz, Beryllium, Asbestfaserstaub, Metallstaub) verursacht werden. Wird alveolengängiger Staub (Partikelgröße < 5 µm) im Gewebe deponiert, sind zwei pulmonale Reaktionsformen möglich.

### Alveoläre Deposition ohne/mit minimaler entzündlicher Reaktion.

Die inhalierten Stäube bilden zunächst lediglich Staubdepots. In der HRCT zeigen sich zentrilobuläre Milchglasnoduli bis 3 mm Durchmesser. Beispiele: zinnhaltiger Staub (Stannose), eisenoxidhaltiger Staub (Siderose).

Im Verlauf – insbesondere bei länger anhaltender kontinuierlicher Exposition – kann aber auch bei diesen Staubexpositionen eine fibrosierende ILD auftreten (z. B. Siderofibrose). Die Annahme, dass es völlig inerte Stäube gibt, wurde mittlerweile verlassen [10].

### Alveoläre Deposition mit nodulärer oder diffuser Bindegewebsbildung.

Im Interstitium deponierte fibrogene Stäube lösen eine chronische entzündliche Reaktion des interstitiellen Lungengewebes aus. Je nach Zusammensetzung des Staubes resultieren in der HRCT dominant noduläre (z. B. Silikose) bzw. dominant retikuläre Bildmuster (z. B. Asbestose), oder es entsteht ein Mischbild aus Milchglas- und retikulärem Muster (z. B. Riesenzellpneumonitis bei Inhalation von Hartmetallstäuben).

Das Risiko, an einer Pneumokoniose zu erkranken, wächst mit steigender Konzentration des einatembaren alveolengängigen Staubes in der Atemluft, mit zunehmendem Gehalt an fibrogenen Substanzen im Staub und der Expositionszeit. Je nach fibrogener Potenz des inhalierten Staubes sowie inhalierter Dosis können die Erkrankungen auch lange nach Expositionsende auftreten (sog. Latenzzeit) und lange nach Expositionsende progredient verlaufen.

## Pneumokoniosen mit dominant nodulärem Muster in der HRCT

### Silikose BK 4101

Der Begriff Silikose bezeichnet in Deutschland synonym eine Gruppe von Erkrankungen, die durch die Inhalation quarzhaltiger Stäube hervorgerufen wird. International wird die Silikose, die durch Inhalation von nahezu reinem Quarzstaub entsteht, von der Mischstaub-Pneumokoniose der Bergleute, die durch Inhalation von quarzhaltigen Staubgemischen entsteht, unterschieden.

Die Erkrankung ist neben der Deposition des Staubes mit nachfolgender Parenchymfibrose durch eine Abtransportstörung des Staubes in den Lymphbahnen gekennzeichnet. Silikotische Noduli finden sich deshalb nicht nur zentrilobulär, sondern auch in den peripheren Lymphbahnen der Lunge im Niveau der viszeralen Pleura. Der letzte Befund ist auch bereits ohne Kenntnis der Arbeitsanamnese hochsuggestiv für das Vorliegen einer Silikose.

Nach klinischen und radiologischen Gesichtspunkten werden die im Folgenden genannten Formen der Silikose unterschieden.

#### Akute Silikose – Silikoproteinose

Dies ist eine sehr seltene, schnell progrediente Erkrankung. Sie kann bei kurzzeitigen sehr hohen Quarzstaubexpositionen ohne geeignete Schutzmaßnahmen entstehen und manifestiert sich in der Regel innerhalb von 3 Jahren nach Expositionsbeginn [21]. In der Literatur werden fokale Milchglasverschattungen, Noduli, Konsolidierungen und Befunde beschrieben die einer Alveolarproteinose (PAP) ähneln. Arbeitsbedingter Auslöser einer PAP ist vorrangig Quarz, aber auch Indiumzinnoxid, und eine Vielzahl weiterer Schadstoffe wird mit der Entstehung einer PAP in Verbindung gebracht [18].

#### Akzelerierte chronische Silikose

Diese Form ist weniger häufig, die Bildmorphologie entspricht der einer chronischen Silikose. Sie tritt typischerweise 4 bis 10 Jahre nach Beginn der Quarzstaubexposition auf und wird durch eine hohe Staubexposition ausgelöst.

#### Unkomplizierte chronische Silikose

**Abb. 1:**
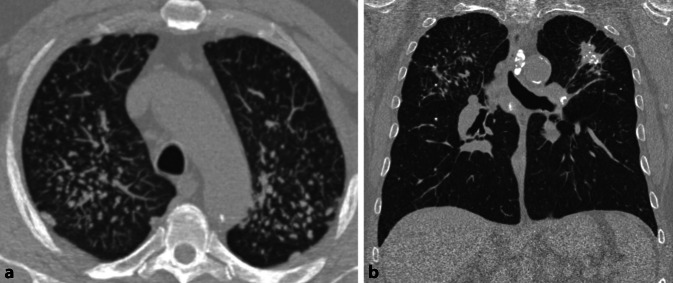
**a** Unkomplizierte Silikose, MIP-Rekonstruktion: Noduli dominant dorsal der Trachealebene und im Niveau der viszeralen Pleura. **b** Übergang in komplizierte Silikose, Konglomeratschatten links und verkalkte Lymphknoten

Hierbei handelt es sich um die häufigste Form der Silikose. Sie entwickelt sich 10 bis 30 Jahre nach Beginn einer Quarzstaubexposition, üblicherweise besteht auch nach Expositionsende ein langsam progredienter Verlauf (Abb. 1a; HRCT-Muster s. Tab. 2).

**Tab. 2:** Befunde der hochauflösenden Computertomographie (HRCT) bei chronischer Silikose

**Unkomplizierte chronische Silikose und akzelerierte Form**	
*Parenchymmuster*	*Lokalisation*
Scharf begrenzte Noduli < 1 –10 mm, auch komplett oder inkomplett verkalkt	Perilymphatisch Staubdepots auch in den Lymphabflusswegen der Pleura Segmente 1, 2, 6 bds. (dorsal der Trachealebene), oft rechts betont
Im Verlauf Koaleszenzen der Noduli möglich im Niveau der viszeralen Pleura tafelbergartige Koaleszenzen möglich	
**Komplizierte chronische Silikose PMF**	
*Parenchymmuster*	*Lokalisation*
Konglomerate häufig oval, Größe wird in der langen Achse bestimmt: A: bis 5 cm, B: > 5 cm < Fläche re OF C: > Fläche rechts OF	OF und MF bds., Orientierung oft parallel zur Thoraxwand
Perinoduläres Emphysem	
**Lymphknoten**	
Vergrößert auch verkalkt (Details s. Tab. 3)	Bihilär und mediastinal
Hiläre Lymphknotenkonglomerate können Stenosen von Bronchien mit Teil‑/Atelektasen der zugehörenden Lungenanteile (meist Mittellappen) und Gefäßstenosen auch mit lokalen Thrombosen verursachen	

#### Komplizierte chronische Silikose (progressive massive Fibrose = PMF)

Durch Koaleszenz silikotischer Noduli entstehen Konsolidierungen, die > 1 cm in der langen Achse (silikotische Schwielen) messen (Abb. 1b).

Sie können eine erhebliche Distorsion des Lungenparenchyms verursachen. In ausgeprägten Fällen kann dabei ein vikariierendes Emphysem anterior und basal entstehen.

#### Lymphknoten bei Silikose

Bei Silikose sind Lymphknoten meist mediastinal und bihilär vergrößert [9]. In ca. 50 % der Fälle finden sich Verkalkungen, wobei die eierschalenartige Verkalkung des Randsinus keineswegs pathognomonisch für die Silikose ist (Tab. 3).

**Tab. 3:** Verkalkungsmuster der Lymphknoten bei Silikose. (Mod. nach [3, 24])

Verkalkungsmuster hilärer und mediastinaler Lymphknoten bei Silikose	
Grobschollig	58–66 %
Diffus	22–24 %
Zentral/exzentrisch	12 %
Eierschalenartig	6–11 %

#### Silikotuberkulose BK 4102

Das Risiko, bei bestehender Silikose an einer Mykobakteriose zu erkranken, ist signifikant erhöht [13]. Hinweise auf eine Tuberkulose sind neu aufgetretene Konsolidierungen, die auch Kavernen aufweisen können, asymmetrische kleine Herde, lokal verdickte bronchovaskuläre Bündel und zentrilobuläre Noduli mit Tree-in-bud-Muster [9, 19].

#### Diffuse interstitielle Fibrose bei Silikose

Insbesondere bei den Mischstaubpneumokoniosen aus dem Kohlebergbau werden bei langjähriger Exposition (unabhängig von einem koinzidenten Tabakrauchabusus) Bildmuster fibrosierender ILD beobachtet [4, 12, 25]. Die Genese ist bislang unklar.

#### Assoziation einer Quarzstaubexposition mit Autoimmunerkrankungen bzw. entzündlich-rheumatischen Erkrankungen

Im Jahr 1953 beschreib Caplan erstmals pulmonale Rheumaknoten („Caplan-Syndrom“) bei Silikose [8]. In den letzten Jahren mehren sich Hinweise auf eine Assoziation einer früheren hohen Quarzstaubexposition mit Autoimmunerkrankungen, wie z. B. Sarkoidose, rheumatoide Arthritis oder einer systemischen Sklerose („Erasmus-Syndrom“) auch ohne Ausbildung einer Silikose [15]. Diese Krankheitsbilder können bislang in Deutschland unfallversicherungsrechtlich nicht anerkannt werden.

### Differenzialdiagnosen zur Silikose

Die Abgrenzung einer Silikose von einer Sarkoidose kann schwierig sein. Beides sind klassische multinoduläre Erkrankungen mit Ober- bzw. Mittelfelddominanz. Beide weisen häufig eine hiläre sowie mediastinale Lymphadenopathie sowie Verkalkungen in den Lymphknoten auf. In der Literatur werden zudem Einzelfälle mit koinzidentem Auftreten beider Erkrankungen beschrieben ([16]; Tab. 4).

**Tab. 4:** Mögliche Anhaltspunkte für eine Differenzierung von Silikose und Sarkoidose

	Silikose	Sarkoidose
Lokalisation in den Lungenlappen	Dominant dorsal Segmente 1, 2 und 6 bds.	Keine dorsale Dominanz
Lokalisation zum Lobulus	Zentrilobulär *und* perilymphatisch Peribronchovaskuläres Interstitium weitgehend ausgespart	Perilymphatisch *Auch* entlang der bronchovaskulären Bündel und der interlobulären Septen
Verlauf	Nie regredient	Regredienz möglich

### Chronische Berylliose BK 1101

Die chronische Berylliose ist eine seltene Berufskrankheit, die hinsichtlich der Symptome, der Histologie und auch des HRCT-Musters nicht sicher von der Sarkoidose zu unterscheiden ist [20].

Bei histologischer Diagnose einer Sarkoidose ist die Erhebung einer qualifizierten Arbeitsanamnese daher unabdingbar, um eine Berylliose nicht zu übersehen. Weist sie auf eine Berylliumexposition hin, verifiziert ein zweimalig positiver Lymphozytenproliferationstest die Diagnose. Arbeitsbedingte Berylliumexpositionen können z. B. im Automobilbau, in der Rüstungsindustrie, im Werkzeug- und Formenbau für den Druckguss, in der Flugzeug- und Weltraumtechnik und in der Keramikindustrie auftreten.

### Talkose

Die Talkose ist eine seltene Pneumokoniose, die in Deutschland nicht als eigenständige Berufskrankheit anerkannt ist (HRCT-Bildmuster der Talkose in Tab. 5).

**Tab. 5:** Befunde der hochauflösenden Computertomographie (HRCT) bei Talkose. (Mod. nach [1])

**Parenchymmuster**	**Lokalisation**
Unscharfe Noduli	Zentrilobulär Zentral und im Lungenmantel Basale Lungenanteile relativ ausgespart
Milchglas fokal	
Verdickungen interlobulärer Septen	
Inhomogene Konsolidierungen mit Talkumdeposition	Keine bevorzugte Lokalisation
**Lymphknoten**	
Erhöhte Dichte durch Talkumdeposition	Mediastinal und hilär nur gering vergrößert

Da Talk, Quarz und Asbest in den gleichen geologischen Gesteinsschichten vorkommen, können je nach Herkunft Verunreinigungen von Talk mit Asbestfasern oder Quarz vorliegen, die eine Silikose bzw. eine asbestfaserbedingte Erkrankung von Pleura und/oder Lunge auslösen können (Bildmuster s. dort; [1, 23]).

## Pneumokoniosen mit dominant retikulärem (netzartigen) Muster in der HRCT

### Asbeststaublungenerkrankung (Asbestose) oder durch Asbeststaub verursachte Erkrankung der Pleura

In Deutschland werden unter der Berufskrankheit Nr. 4103 die benignen Erkrankungen Asbeststaublungenerkrankung (Asbestose) und die durch Asbestfaserstaub verursachte Erkrankung der Pleura zusammengefasst.

#### Asbeststaublungenerkrankung – Asbestose BK 4103

Bei Asbestfaserinhalation kann sich eine nichtgranulomatöse Fibrose der Lunge entwickeln. Die Latenzzeit zwischen der Exposition und dem Auftreten einer ILD beträgt in der Regel mehr als 20 Jahre. Die Schwere der Erkrankung nimmt mit der Höhe der Staubexposition zu.

Initial besteht eine peribronchioläre Fibrose mit nachfolgender Beteiligung der Alveolarkanäle, es folgt ein Kollaps der Alveolen. Bei hoher Staubexposition kann sich eine schnell progrediente Kombination aus alveolärem Kollaps und kollagener Fibrose entwickeln, die die Alveolarlumina ausfüllt, UIP-artige Muster (gewöhnliche interstitielle Pneumonie) können entstehen. Bei niedrigeren Staubexpositionen kann im Langzeitverlauf ebenfalls eine fibrosierende Erkrankung entstehen, die allerdings eher ein unspezifisches- oder NSIP-artiges Muster (nicht spezifische interstitielle Pneumonie) aufweist [1, 5].

**Abb. 2:**
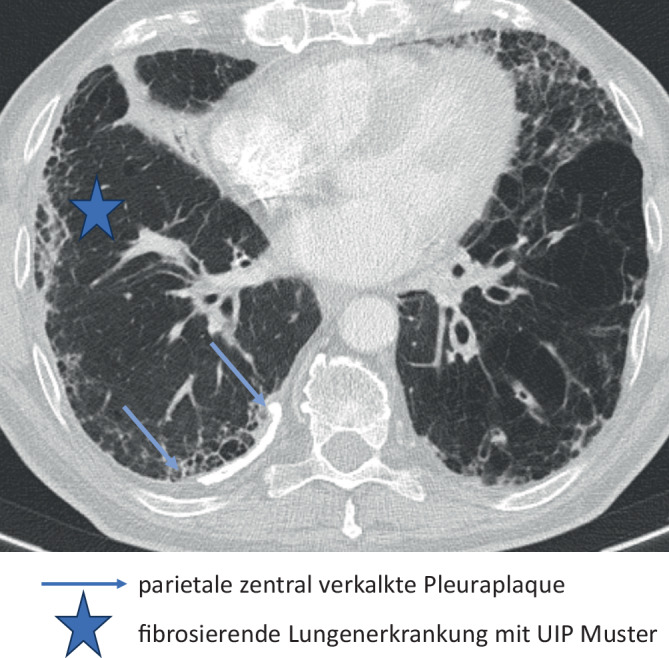
Asbestfaserbedingte Erkrankung von Lunge und Pleura

In der HRCT finden sich heute in Industrieländern überwiegend Parenchymmuster, die einem Initialstadium entsprechen. Das radiologische Bild ist zu diesem Zeitpunkt unspezifisch und nicht eindeutig einer Fibrose zuzuordnen (Tab. 6). Bei einer fortgeschrittenen Erkrankung sind differenzialdiagnostisch auch andere ILD mit UIP- oder NSIP-artigem Muster in Erwägung zu ziehen (Abb. 2). Bei bekannter Asbestfaserexposition kann in Deutschland eine fibrosierende Lungenerkrankung unfallversicherungsrechtlich dann mit überwiegender Wahrscheinlichkeit einer Asbestose zugeordnet werden, wenn bildmorphologische Kriterien einer asbestfaserbedingten Erkrankung der Pleura vorliegen und anderweitige Ursachen der pleuralen Verdickungen ausgeschlossen werden können.

**Tab. 6:** Befunde der hochauflösenden Computertomographie (HRCT) bei Asbestose. (Mod. nach [1])

**Initialstadium der Asbestose**	
*Parenchymmuster*	*Lokalisation*
Noduli	Zentrilobulär; v. a. im Lungenmantel dorsal/basal
Noduli verschmelzen zu kurvilinearen Verdichtungen	Im Lungenmantel parallel zur Thoraxwand dorsal/basal
**Progrediente Form der Asbestose**	
Intra- und interlobuläre Retikulationen	Vor allem im Lungenmantel der Unterlappen Segmente 9 und 10, auch Mittellappen und Lingula basal
Traktionsbronchiektasen	
Honeycombing

#### Benigne asbestfaserbedingte Erkrankung der Pleura BK 4103

Charakteristisch sind umschriebene spindel- oder tafelbergartige Areale mit einer hyalinen Fibrose der Pleura parietalis (sog. Pleuraplaques), die auch bei niedrigen Staubexpositionen ohne begleitende ILD auftreten können. Bei beidseitigem Auftreten sind sie bei Ausschluss anderweitiger Ursachen pathognomonisch. Eine Asbestfaserinhalation kann auch eine umschriebene oder langstreckige Verdickung der Pleura visceralis, eine Pleuritis, eine Hyalinosis complicata sowie Rundatelektasen induzieren (Tab. 7).

**Tab. 7:** Pleurabefunde nach Asbestfaserinhalation

**Parietale Pleuraverdickung**	**Lokalisation**
Tafelbergartig/spindelförmig, auch verkalkt	OF/MF anterior/lateral, MF/UF lateral/dorsal paravertebral, ZF v. a. zentral, mediastinal *ohne Beteiligung des Sinus*
**Viszerale Pleuraverdickung**	**Lokalisation**
Tafelbergartig/spindelförmig, auch verkalkt, davon ausgehende pleuropulmonale Narbenstränge	Siehe oben; sehr selten an Lappenspalten, ohne pleuropulmonale Narbenstränge
Hyalinosis complicata: nach rezidivierendem Pleuraerguss	Basal *mit Beteiligung des Sinus*
Rundatelektase: nach rezidivierendem Pleuraerguss (Kombination aus narbig geschrumpfter Pleura und darin „gefesselter“ Lunge)	Ubiquitär
Pleuraerguss	

### ILD auf dem Boden der Inhalation von Metallstäuben

Die Inhalation von Metallstäuben und ihrer Dämpfe kann fibrosierende ILD auslösen. Vor allem finden sich UIP- bzw. NSIP-artige Muster, aber auch Bildmuster granulomatöser Erkrankungen. Die HRCT-Befunde lassen keinen sicheren Rückschluss auf das inhalierte Agens zu.

### Aluminose BK 4106

Aluminiumstäube können sowohl toxische Reaktionen als auch eine interstitielle Lungenerkrankung hervorrufen. Die Pathogenese der fibrosierenden ILD ist nach wie vor unklar. Das Bildmuster im HRCT ist vielfältig (Tab. 8). Die Diagnosestellung wird durch hohe Al-Konzentrationen in Plasma und Urin erleichtert.

**Tab. 8:** Befunde der hochauflösenden Computertomographie (HRCT) bei Aluminose. (Mod. nach [1, 17])

**Initialstadium der Aluminose**	
*Parenchymmuster*	*Lokalisation*
Unscharfe Noduli	Zentrilobulär, dominant in den Oberlappen
**Progrediente Form der Aluminose**	
Fokal Milchglas	Dominant in den Oberlappen, in fortgeschrittenen Fällen auch Mittel- und Unterlappen
Intra- und interlobuläre Retikulationen	
Honeycombing	
Emphysem bullös im Lungenmantel und vikariierend	
**Lymphknoten**	
Angehobene Dichte durch Aluminiumdeposition	Hilär und mediastinal

### Lungenfibrose bei Exposition gegenüber Hartmetallstäuben BK 4107

Der Begriff Hartmetall wird in der Regel für eine Legierung aus Wolfram, Kohlenstoff und Kobalt verwendet, der gelegentlich kleine Mengen anderer Metalle wie Titan, Tantal, Nickel und Chrom zugesetzt werden. Die Hartmetall-Pneumokoniose kann sowohl bei der Herstellung als auch bei der Nachbearbeitung entstehen. Das HRCT-Bild ist sehr variabel, es kann einem UIP- bzw. NSIP-artigen Muster, aber auch dem fibrosierenden Typ der Hypersensitivitätspneumonitis oder der Sarkoidose ähneln. Sowohl Oberlappen- als auch Unterlappen-dominante Verläufe sind beschrieben. Langsames Fortschreiten sowie auch rapide progressive Verläufe sind bekannt [1].

### Neue seltene arbeitsbedingte Lungenerkrankungen

In den letzten Jahren sind weltweit neue arbeitsbedingte Erkrankungen der terminalen Luftwege und des Lungenparenchyms bekannt geworden. Sie spielen in Deutschland bisher keine Rolle. Unter anderem werden in der Herstellung von z. B. Convenience-Produkten wie aromatisiertem Popcorn, Textilien und in der Elektronikindustrie Werkstoffe verwendet, deren arbeitsbedingte Inhalation eine ILD auslösen kann (Tab. 9; [26]).

**Tab. 9:** Beispiele neuerer arbeitsbedingter interstitieller Lungenerkrankungen

	Schadstoff	HRCT-Muster
Aromatisierung von Lebensmitteln z. B. Popcorn	Diacetyl und Ersatzstoffe	Inhomogene Lungendichte i. S. von Airtrapping
Verarbeitung von Indium	Indiumzinnoxid	Unscharfe zentrilobuläre Noduli, Milchglas, Traktionsbronchiektasen, Crazy-Paving-Pattern
Verarbeitung von Nylonfasen	Polyamid Fasern	Airtrapping, Mikronoduli, fleckförmige Konsolidierungen, Honeycombing
Herstellung/Verarbeitung von Nanopartikeln	Nanopartikel	Fleckförmig Milchglas, unscharfe zentrilobuläre Noduli, Traktionsbronchiektasen

## Fazit für die Praxis

- Zur differenzialdiagnostischen Einordnung einer interstitiellen Lungenerkrankung (ILD) ist die Berufsanamnese unabdingbar!
- Insbesondere bei fibrosierenden ILD auch an arbeitsbedingte Erkrankungen denken!
- Bei fibrosierender ILD unbedingt die Pleura beachten! Pleuraplaques werden häufig in Unkenntnis der Anamnese als postentzündlich eingeordnet.
- Bildmuster von Silikose und Sarkoidose können sich ähneln, Arbeitsanamnese und Verlauf helfen bei der Differenzierung.

## References

[1] Akira M (2021) Imaging diagnosis of classical and new pneumoconiosis: predominant reticular HRCT pattern. Insights Imaging 12:33 33689008 10.1186/s13244-021-00966-y PMC7947097

[2] Akira M, Suganuma N (2014) Acute and subacute chemical-induced lung injuries: HRCT findings. Eur J Radiol 83:1461–1469 24853247 10.1016/j.ejrad.2014.04.024

[3] Antao V C, et al (2005): High-resolution CT in silicosis: correlation with radiographic findings and functional impairment. J Comput Assist Tomogr. May-Jun;29(3):350–6. 10.1097/01.rct.0000160424.56261.bc 15891506

[4] Arakawa H, et al (2007): Chronic Interstitial Pneumonia in Silicosis and Mix-Dust Pneumoconiosis Its Prevalence and Comparison of CT Findings with Idiopathic Pulmonary Fibrosis. Chest 131(6) 1870–1876 10.1378/chest.06-2553 17400659

[5] Attanoos RL (2010) Asbestos-Related Lung Disease. Surg Pathol 3:109–127 10.1016/j.path.2010.04.003 26839029

[6] DGUV Berufskrankheiten Liste: www.dguv.de/bk-info/allgemein/bk-liste/index.jsp

[7] Blanc PD et al (2019) The Occupational Burden of Nonmalignant Respiratory Diseases. An Official American Thoracic Society and European Respiratory Society Statement. Am J Respir Crit Care Med 199:1312–1334 31149852 10.1164/rccm.201904-0717ST PMC6543721

[8] Caplan A (1953) Certain unusual radiological appearances in the chest of coalminers suffering from rheumatoid arthritis. Thorax 8:29–37 13038735 10.1136/thx.8.1.29 PMC1019224

[9] Chong S et al (2016) Pneumoconiosis: comparison of imaging and pathologic findings. Radiographics 26(1):59–77 10.1148/rg.261055070 16418244

[10] Churg A (2006) Responses of the Respiratory System to Inhaled Agents (Airways, Lung and Pleura). in: Gevenois PA and De Vuyst P (Eds) Imaging of occupational and Environmental Disorders of the Chest. Springer Sci Bus Media Berl: 13–30

[11] Cohen RA et al (2023) Global Trends in Occupational Lung Disease. Semin Respir Crit Care Med 44:317–226 37072021 10.1055/s-0043-1766117

[12] Cox CW et al (2014) State of the art: Imaging of occupational lung disease. Radiology 270(3):681–696 24568704 10.1148/radiol.13121415

[13] Ehrlich R et al (2021) The association between silica exposure, silicosis and tuberculosis: a systematic review and meta-analysis. Bmc Public Health 21:953 34016067 10.1186/s12889-021-10711-1 PMC8136154

[14] Hering KG et al (2014) Update: Standardisierte CT-/HRCT-Klassifikation der Bundesrepublik Deutschland für arbeits- und umweltbedingte Thoraxerkrankungen. Radiologe 54:363–384 24737105 10.1007/s00117-014-2674-y

[15] Hoy RF, Chambers DC (2020) Silica-related diseases in the modern world. Allergy 75:2785–2797 10.1111/all.14202 31989662

[16] Hübener E et al (1986) Coincidence of silicosis and sarcoidosis. 2. Relations between silicosis and sarcoidosis as well as forensic consequences. Z Erkr Atmungsorgane 166(2):186–193 3716495

[17] Kraus T et al (2006) Aluminosis—detection of an almost forgotten disease with HRCT. J Occup Med Toxicol 1:4 16722569 10.1186/1745-6673-1-4 PMC1436008

[18] Kumar A, Cummings C (2021) Pulmonary Alveolar Proteinosis Secondary to occupational exposure. Curr Pulmonol Rep 10:30–39 10.1007/s13665-021-00267-1

[19] Lanzafame M, Vento S (2021) Mini-review: Silico-tuberculosis. J Clin Tuberc Other Mycobact Dis 23:100218 33598569 10.1016/j.jctube.2021.100218 PMC7868994

[20] Lynch DA (2006) Imaging of beryllium-related diseases. In: Gevenois PA, De Vuyst P (Hrsg) Imaging of occupational and Environmental Disorders of the Chest. Springer Science & Business Media. Berlin:, S 249–256

[21] Marchiori E et al (2007) Silicoproteinosis: High-Resolution CT Findings in 13 Patients. AJR Am J Roentgenol 189(6):1402–1406 18029877 10.2214/AJR.07.2402

[22] Nagel H et al (2017) Protokollempfehlungen der AG DRauE zur Durchführung von Low-Dose-Volumen-HRCT-Untersuchungen der Lunge. Fortschr. Röntengstr, Bd. 189, S 553–575

[23] Neumann V et al (2011) Fallbericht zu einer seltenen Berufskrankheit: Eine zu Lebzeiten nicht anerkannte Talkose. Pneumologie 65:471–476 21412706 10.1055/s-0030-1256286

[24] Ooi CGC et al (2003) The relationship between mediastinal lymph node attenuation with parenchymal lung parameters in silicosis. Int J Tuberc Lung Dis 7(12):1199–1206 14677896

[25] Petsonk EL et al (2013) Coal Mine Dust Lung Disease: New Lessons from an Old Exposure. Am J Respir Crit Care Med 187:1178–1185 23590267 10.1164/rccm.201301-0042CI

[26] Sauler M et al (2012) Newly Recognized Occupational and Environmental Causes of Chronic Terminal Airways and Parenchymal Lung Disease. Clin Chest Med 33(4):667–680 23153608 10.1016/j.ccm.2012.09.002 PMC3515663

[27] Akira M, Suganuma N (2023) Imaging diagnosis of pneumoconiosis with predominant nodular pattern: HRCT and pathologic findings. Clin Imaging 97:28–33 36878176 10.1016/j.clinimag.2023.02.010

[28] Kreuter M et al (2023) S1-Leitlinie Interdisziplinäre Diagnostik interstitieller Lungenerkrankungen im Erwachsenenalter. Pneumologie 77:269–302 36977470 10.1055/a-2017-8971

